# P01.32. Can homeopathic verum and placebo globules be distinguished by UV spectroscopy?

**DOI:** 10.1186/1472-6882-12-S1-P32

**Published:** 2012-06-12

**Authors:** U Wolf, S Klein

**Affiliations:** 1 University of Bern, Institute of Complementary Medicine KIKOM, Bern, Switzerland

## Purpose

Homeopathic preparations are used in homeopathy and anthroposophically extended medicine. Previous studies described differences in UV transmission between homeopathic preparations of CuSO_4_ and controls. The aim of the present study was to investigate whether statistically significant differences can be found between homeopathic verum and placebo globules by UV spectroscopy.

## Methods

Verum (aconitum 30c, calcium carbonate/quercus e cortice) and placebo globules used in two previous clinical trials were dissolved in distilled water at 10mg/ml 20-23 hours prior to the measurements. Absorbance was measured at 190 – 340nm with a Shimadzu UV-1800 double beam spectrophotometer. Duplicates of each sample were measured in a randomized order 4 times on each of the 5 measurement days. To correct for differences between measurement days, average absorbance of all samples on one day was deduced from absorbance of the individual samples. The Kruskal-Wallis test was used to determine group differences between the samples, and finally the coding of the samples was revealed.

## Results

First analysis showed significant differences (p≤0.05) in average UV absorbance at 200 – 290nm between the samples and a tendency of a correlation (p≤0.1) between absorbance and globule weight. More results will be presented at the conference.

## Conclusion

Since the absorbance of the samples at the wavelengths between 200 and 290nm was small, a number of aspects had to be considered and should be corrected for if they are present when performing UV spectroscopy on homeopathic globules: (1) Exact weighing of the globules; (2) Measurement error of the spectrophotometer at small absorbances; (3) Drift of the spectrophotometer during a measurement day; (4) Differences between measurement days.

The question remains what caused the differences in absorbance found in these experiments; the use of the original material for the production of the verum globules, differences in the production of verum and placebo globules, or other context factors.

